# Supplementary material for: Ceruloplasmin Interferes with the Assessment of Blood Lipid Hydroperoxide Content in Small Ruminants
Source: Antioxidants (Basel). 2023 Mar 12;12(3):701. doi: 10.3390/antiox12030701 (PMC10045310; doi:10.3390/antiox12030701)
Supplement: Supplementary file 1 [file antioxidants-12-00701-s001.zip › antioxidants-2221314-supplementary.pdf]

Supplemental Materials

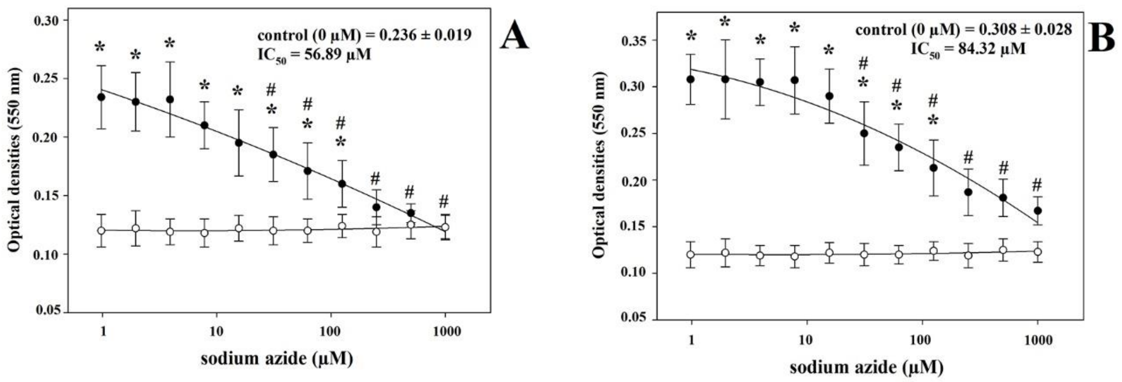

Figure S1. Dose-dependent effect of sodium azide treatment in adult goat (A) and sheep (B) blood serum samples, as assessed using a ROMs assay (filled circles). Blank abs: optical densities of blank wells treated with the same sodium azide concentration as the blood serum samples (empty circles). Mean values with the superscript # and * show statistically significant differences in comparison with the lowest (0 μM) and the highest (1000 μM) sodium azide concentrations, respectively.
